# Scale Determinants of Fiscal Investment in Geological Exploration: Evidence from China

**DOI:** 10.1371/journal.pone.0076656

**Published:** 2013-10-29

**Authors:** Linna Lu, Yalin Lei

**Affiliations:** 1 School of Humanities and Economic Management, China University of Geosciences, Beijing, China; 2 Chinese Academy of Land and Resource Economics, Beijing, China; University of Florida, United States of America

## Abstract

With the continued growth in demand for mineral resources and China's efforts in increasing investment in geological prospecting, fiscal investment in geological exploration becomes a research hotspot. This paper examines the yearly relationship among fiscal investment in geological exploration of the current term, that of the last term and prices of mining rights over the period 1999–2009. Hines and Catephores' investment acceleration model is applied to describe the scale determinants of fiscal investment in geological exploration which are value-added of mining rights, value of mining rights and fiscal investment in the last term. The results indicate that when value-added of mining rights, value of mining rights or fiscal investment in the last term moves at 1 unit, fiscal investment in the current term will move 0.381, 1.094 or 0.907 units respectively. In order to determine the scale of fiscal investment in geological exploration for the current year, the Chinese government should take fiscal investment in geological exploration for the last year and the capital stock of the previous investments into account. In practice, combination of government fiscal investment in geological exploration with its performance evaluation can create a virtuous circle of capital management mechanism.

## Introduction

China's investment mechanism on geological exploration contributes to a special investment scenario that fiscal investment occupies a substantial proportion of the total investment. For instance, fiscal investment in geological exploration accounts for 15.4% in 2009 [1]. The investment mechanism of China's geological exploration differs from that of the western market economies. In western market economies, central government usually only invests on public geological survey while private funds invest in commercial mineral exploration. Chinese government invests in public geological survey and commercial mineral exploration as well for it sets up geological exploration funds to share the risk of some of the commercial investors for China has not established risk capital market of exploration yet [2]. Therefore, the new mechanism of geological exploration investment is being implemented in practice, which is “public capital goes first, exploration funds play a convergent role, commercial capital follows up, be fully equipped to exploration and make a breakthrough” [3].

Most literatures on geological exploration investment shed light on the economic impact evaluation of the total investment [4], [5]; however, scarce literatures practically analyze the internal relations of fiscal investment in geological exploration. Some observers notice that China's fiscal funds investing in geological exploration have been declining gradually [5]–[8] and private capital has been entering that field [6], [9]. Despite some discussion on fiscal investments scale on geological exploration [10]–[12], there is still little research on the scale determinants and relevant policies. In the long run, since China's fiscal funds will still play an important role in geological exploration investment due to its centralized governance structure and the concern of sharing risk for private investors, the corresponding fiscal policies need to be further studied.

On the basis of the literature review, the investment acceleration model is applied to analyze the scale determinants of fiscal investment in geological exploration and corresponding policy suggestions have been given with future research directions being proposed.

## Literature Review

Keynes [13] created a true sense of research on public investment and economic growth in “General Theory”. He believes that for the role of monetary policy is highly weak, the state should adopt fiscal policies to strengthen the main socio-economic intervention and the range of expenditure should be expanded to economic spending such as infrastructure in addition to traditional government spending. For issues related to fiscal investment, research literature can be inspected from the following aspects, such as capital structure, size, effects and institutional issues [14]–[21]. However, few studies attempt to determine the optimal scale of fiscal expenditure by economic growth [22]. Barro [16] firstly developed the theoretical framework of optimal scale of government expenditure via endogenous growth model. Further, Barro proposed the “Barro Rule”-the government services are “optimally provided” when the marginal product of government consumption equals unity. Steven P. Cassou and Kevin J. Lansing [23] develops a quantitative theoretical model for the optimal provision of public capital. Using the theoretical framework of Barro, the optimal scale of government services and fiscal expenditure in the process of economic growth was investigated [22], [24]–[25]. Using panel data, the “Barro rule” for 118 countries and European economies has been examined respectively [24]–[25]. G. Karras finds that all government services are productive in the sense that their marginal product is positive and significantly different from zero [25]. Gunalp and Guzhan [22], using annual data from 1990 to 2001 for 20 transition countries, find empirical evidence supporting the hypotheses that government services are productive, and the optimal government scale is estimated to be 17.3 percent (±3 percent) for the average transition country.

Though research on domestic fiscal investment started late, a number of fiscal policy recommendations under China's national conditions have been created [26]–[33]. Xiaoming LI [34] developed a two-region game theoretical model to argue that the prospective financial reforms will subject local governments' investment driving to the indirect regulations of monetary policy. Yehua WEI [35] investigates China's changing fiscal system and its impact on uneven regional development. S. MA and C. SUN [36] developed an endogenous growth model to estimate the optimal scale of fiscal expenditure in China. His final conclusion is that the total optimal scale of fiscal expenditure is 24% of GDP. Liyong WANG and Wei GAO [37] use the Markov-switching model to test the nonlinear effects of government expenditure and taxes on private consumption in China. The results show that fiscal policy in China has a significantly nonlinear effect. Rui ZHANG and Zhong-fu LI [38] employ ‘Barro rule’ and Karras's theoretic framework, applies C-D production function model and investigates the optimal scale of rural fiscal expenditure in China. Yihua YU, Li ZHANG, Fanghua LI and Xinye ZHENG [39] use cross-sectional data of 242 Chinese cities in 2005 to explore the major factors contributing to the decline of public investment.

China's current fiscal system is largely decentralized while its governance structure is rather centralized with strong top-down mandates and a homogeneous governance structure, thereby creating a healthy investment environment for the nonfarm sector to grow [40]–[47]. The decentralized fiscal system promotes the fiscal funds to invest in geological exploration for the local governments would like to invest and boost the local mining industry. Because of the special investment mechanism of geological exploration, the central and local fiscal funds invest in public geological survey and commercial mineral exploration as well [5], [39]. Policies of fiscal investment in geological exploration arouse observers' interests. Ziran Z. [12] details the policies and actions of the Chinese Government on the exploration and development of mineral resources in the western region in support of the WDI (The Western Development Initiative) region. He advocates that the scale of central fiscal transfer disbursement to the WDI region will be expanded. Yunzhong LIU, Xiaocai YOU, Li GAO and Jinghua CHENG [8] suggests that public mineral geological work, supported by national fiscal funds, affects the national economy through the needs of mineral resources and proposes to adopt public investment policies of mineral geology in Chinese scenario. Unfortunately, there is little research, theoretically or experimentally, on the scale determinants of China's fiscal investment in geological exploration, which is the main target of this paper. Since there is a tendency of performance-oriented fiscal policies in fiscal managerial field [48]–[51], as an investment behavior, fiscal investment has something in common with investments in other fields. As a result, investment model in other fields (see [52]–[58]) can be used to discuss the fiscal investment policies in geological exploration.

## Methodology

### Model design

Hines and Catephores [58] proposed the investment acceleration model using the latest information to determine the expected value of capital stock, based on the latest information on the level of output rather than the actual level of output remains to be seen. The scenario of fiscal investment in geological exploration in China is similar with the scenario that the Hines and Catephores' model describes. The model includes the latest information on the level of output, such as output capital value-added and output capital for each term which can annotate the value-added and the value of mining rights. That can account for the reason why we choose the model to research on determinants of fiscal investment in geological exploration in China.

According to [58], the equations fitted to U.K. quarterly data for the period 1956–67 by the method of ordinary least squares are as follows.
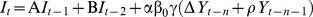
(1)

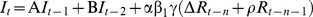
(2)

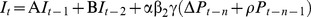
(3)

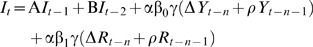
(4)

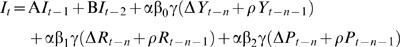
(5)

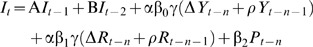
(6)



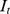
 is the investment, that is gross fixed capital formation in manufacturing industry. 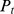 is gross trading profits of companies. 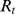 is a quarterly average of the flat yield on 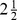 percent consults. 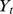 is an index of output in manufacturing, used as a proxy for demand. Experiments with the data suggested that 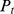 and 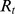 are unsatisfactory variables. As a result, equation (1) can be used as an original model.

The model utilized in this paper is derived in Hines and Catephores's model and can be described as follows.
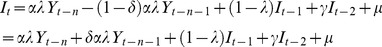
(7)


The simplified form is as follows.
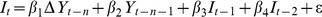
(8)


Where 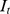 = investment for each term, 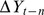 = output capital value-added for each term, 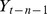 = output capital for each term, 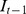 = investment for the last term, 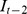 = investment for the last second term. 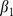, 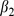, 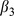, 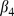 denote the impact parameter of 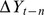, 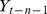, 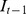, 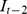 respectively. 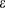 denotes random disturbance term.

### Data Description

In this paper, data from 1999 to 2009 comes from [1]. Hong Kong, Macao and Taiwan Province are not included in those national statistics. In equation (8), 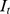 represents fiscal investment in geological exploration at industry level, 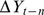 denotes output capital (denotes prices of mining rights) value-added for previous term, 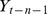 denotes output capital (denotes prices of mining rights) for previous term, and 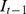 as well as 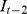 represent fiscal investment in geological exploration for the last term and the last second term respectively. The mining rights include prospecting and mining rights. Since there is only data on prices of mining rights which are the collaborative outcome of fiscal and social funds, we can obtain separate data on prices of mining rights which are the outcome of fiscal funds by multiplying the total prices of mining rights with the percentage of fiscal funds. Here the premise needs to be set beforehand, that is, the investment efficiency of fiscal funds is the same as social funds for the funds have the same properties as currency and capital. Fiscal funds include the fiscal funds from central government and local governments. The above data is on the base year 1999, taking inflation into account, deflated with the GDP index.

### Data Analysis

This statistical analysis, with SAS 8.11 software and OLS method, can estimate the Hines and Catephores investment acceleration model considering 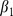, 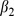, 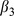, 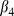. The regression equation is obtained as follows:
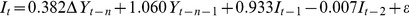
(9)

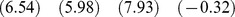


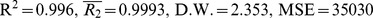



The P-values of variables 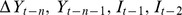 are 0.0006, 0.0010, 0.0002, 0.7596, which indicates the first three variables are significant while 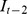 is not.

**Figure 1:**
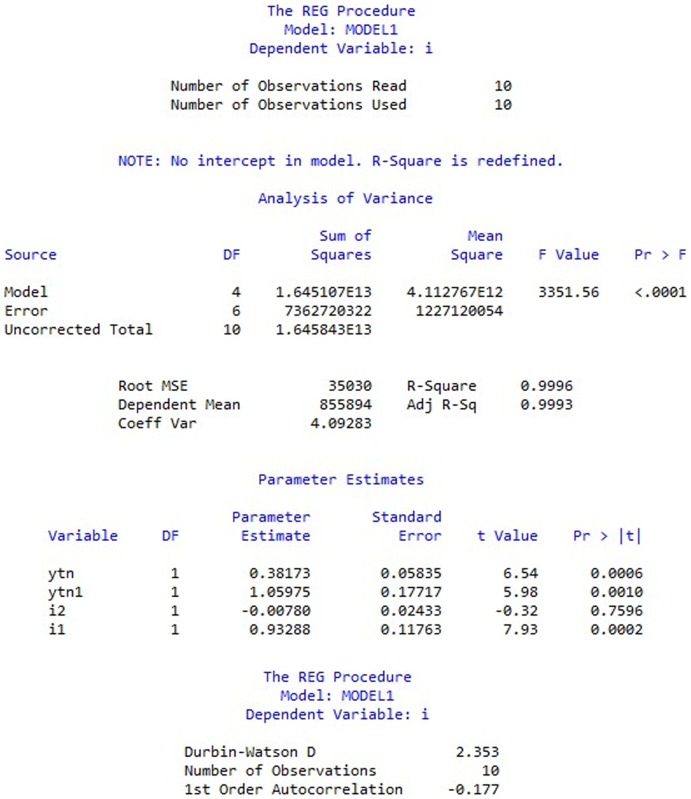
Estimation of investment function model. Estimation of investment function model includes analysis of variance and parameter estimates towards variables 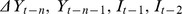 (data from [1]).

From the above result (as in Fig. 1), it can be inferred that the model is significant while 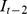 is not significant. Why the model is significant while one of the variables is not? Simply removing 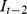 from the model is not logical. The insignificance of 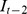 is possibly caused by other variables. Which of the variables should we choose and maintain while removing the rest? Therefore, it is needed to compare the difference of variables' significant contribution and discuss the reasonability of the model by stepwise regression.

**Figure 2:**
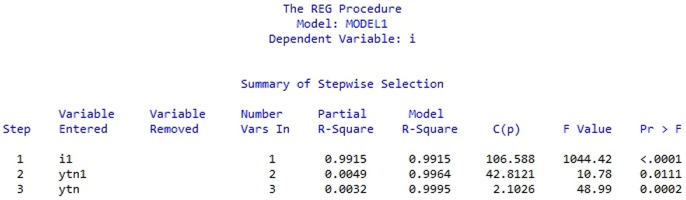
Stepwise regression. Stepwise regression is conducted among four variables 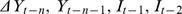 (data from [1]).

Through the stepwise regression (as in Figure 2), we can figure out that 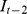 is the least significant variable among the four ones. It needs to be removed to ensure the reasonability of the model. Then the model can be adjusted as follows.

**Figure 3:**
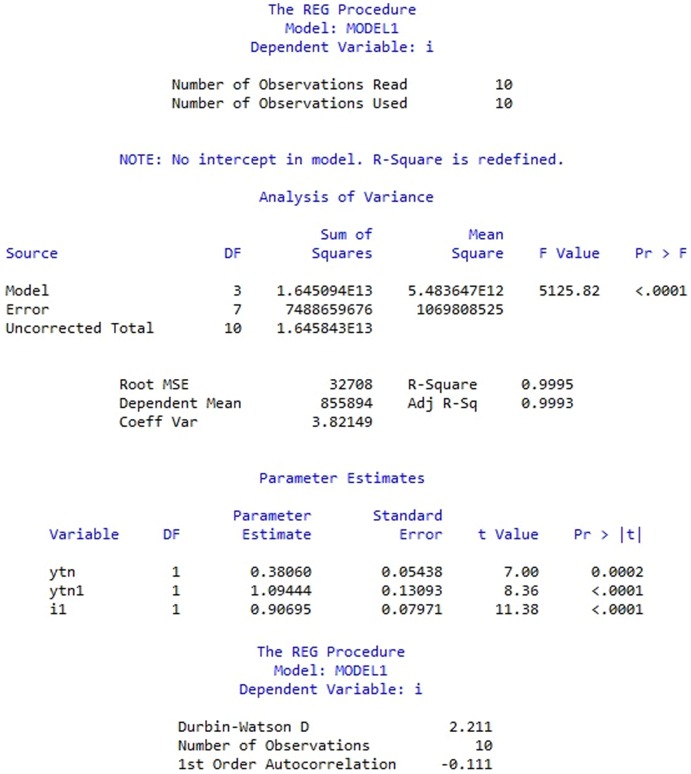
Estimation of adjusted investment function model after removing 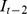. Estimation of adjusted investment function model includes analysis of variance and parameter estimates towards variables 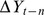, 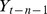 and 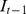 (data from [1]).

After removing 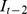, the model is significant at the significant level of 10% (as in Fig. 3). The adjusted regression equation is obtained as follows:
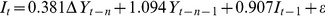
(10)

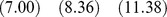


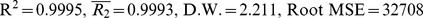



Four parameters in the equation (10) at the 5% significance level were significant, reflecting that value-added of mining rights, value of mining rights and fiscal investment in the last term have long-run equilibrium relationship and positive correlation with fiscal investment in the current term. Value of mining rights and fiscal investment in the last term has greater influence on fiscal investment in the current term than value-added of mining rights. The coefficient of 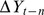 is 0.381, which indicates when value-added of mining rights in the last term moves 1 unit, fiscal investment in the current term will move 0.381 units positively. Similarly, when value of mining rights in the last term or fiscal investment in the last term moves 1 unit, fiscal investment in the current term will move 1.094 or 0.907 units respectively.

The equation (10) describes the scale determinants of fiscal investment in geological exploration, which are value-added of mining rights, value of mining rights in the last term and fiscal investment in the last term. Among those three determinants, value-added of mining rights and value of mining rights in the last term reflect the monetary value attribute and characteristics of investment behavior, and the fiscal investment in the last term reflects historical retrospective which is consistent with China's current fiscal management method.

## Conclusion, Discussion and Future Research Directions

### Conclusion

The paper applies Hines and G. Catephores' investment acceleration model with the latest information to determine the scale of fiscal investment in geological exploration. The conclusions can be drawn as follows. (1) Fiscal investment in the current term will move 0.381 units positively when value-added of mining rights in the last term moves 1 unit. (2) Fiscal investment in the current term will move 1.094 units correspondingly when value of mining rights in the last term moves 1 unit. (3) Fiscal investment in the current term will move 0.907 units correspondingly when fiscal investment in the last term moves 1 unit.

### Discussion

Because of China's unique investment mechanism of geological exploration, public finance not only needs to bear the cost of public geological work, but also needs to give financial and administrative support to the growing commercial geological work accompanied by market failures, economic externalities, presence of incomplete markets. With the injection of fiscal funds, pulling effect of social capital into the geological survey and mining industry is obvious [59]. Volume of social capital in geological survey is enormous, indicating that the future profit potential from geological prospecting and exploration has a strong attraction to social capital and social capital is willing to invest in geological prospecting and following mining industry. For the volume of fiscal investment in geological work is more stable than that of social capital and social capital is usually pulled by fiscal funds, one of the critical methods to strengthen the budget management of the geological exploration investment is to determine the reasonable size of fiscal investment in geological exploration. The conclusions of the paper can be beneficial to work out the fiscal investment budget for geological exploration, which reveals the following:

1) Define budget implementation of fiscal investment in geological exploration in the previous year as a factor to determine financial investment in geological exploration in the current year.

People's Republic of China's implementation of the national budget in 2010 and national budget in 2011 (Draft) [60] provides the overall requirements of financial budgeting and financial work. In 2011, for example, China continued to implement the proactive fiscal policy, to make efforts in optimizing the investment structure, to strengthen the weak points in economic and social development, to support economic restructuring and coordinated regional development and to promote economic development mode's alteration. In budget implementation of 2010, land resources and meteorological services expenditures, 45.489 billion RMB, rose up by 22.9 percent than that of 2009, in order to enhance mineral exploration, mine geology and environmental recovery and conservation and comprehensive utilization of mineral resources. It can be inferred that increasing the state's geological exploration input is an important measure of fiscal investment in recent years, which effectively promotes the prosperity of mining and economic development. Appropriate scale of investment is not only the scale that adapts to economic, technological and social development but also the one that can be afforded by the government in a period of time. For geological exploration is performance-oriented, in order to ensure appropriate investment in geological exploration, it is particularly necessary to determine a reasonable investment budget. Model (10) shows that, the amount of financial investment in a geological exploration for the last term is an important basis to determine the amount for the current term, which is consistent with our current budget system. On March 22,1994, the second meeting of the Eighth National People's Congress adopted “Budget Law of the People's Republic”, which indicates that the budget year is from January 1, to December 31 in the Gregorian calendar. The central budget and local government budgets at all levels, should refer to the budget implementation of the previous year and revenue forecast of the current year.

2) Define the capital stock of the previous investment in geological exploration as a factor to determine the current fiscal investment

Model (10) shows that mining output levels, signified by mining rights' prices and value-added, reflect the latest expectations of capital stock, which should also be included in the fiscal budget considerations. Fiscal funds for geological exploration come from “one tax, two prices, three charges” (i.e., resource tax, the prices of prospecting and mining rights, compensation fee of mineral resources, exploration royalties and mining royalties), the geological survey fund and other special charges. The capital stock of fiscal investment in geological exploration, part of prospecting and mining rights' prices, participates in the new round of investment activities on geological exploration, which forms snowballing financial cycle model. Therefore, considering the historical output levels of investment can reflect the funds' acceleration effect and dynamic changes of capital stock in long-term trends, to determine the reasonable scale of fiscal investment in geological exploration.

3) Combination of government fiscal investment in geological exploration with its performance evaluation can help form a virtuous circle of capital management mechanism.

From the perspective of China's current budget making of departments at all levels, while budget preparing at the beginning of period, staffing, public funds and project funds should be taken into consideration, which are not easy to be connected with prices of mining rights. Only under the premise of evaluating the output of fiscal investment, prices of mining rights can be considered as an indicator of preparing the budget. That can provide us with a new approach to prepare fiscal budget of investment in geological exploration. Higher prices of mining rights means better performance of fiscal investment in geological exploration, which suggests that the government shall invest more and make a larger budget. Combination of performance evaluation of fiscal investment with budget preparation can make high efficiency of fiscal funds, which can create a virtuous circle of capital management mechanism.

In practice, value of mining rights affects the amount of investment while the dynamic market and government behavior also has something to do with the latter. The dynamic market and government behavior can cause the price change of mineral resources and fluctuation of mining rights, which can be embodied in the change of value and value-added of mining rights. Those factors have been already taken into account in investment acceleration model.

### Future research directions

This study is based on fiscal investment in geological exploration at industry level. In order to examine the problem more comprehensively, we have three main directions in further studies: (1) To measure the influence of the dynamic market and government behavior on the fiscal investment in geological exploration respectively can rationalize the investment mechanism. (2) To discuss the mechanism of budget implementation of fiscal investment in geological exploration specifically can ensure the validity of the fiscal policy. (3) To discuss the scale of fiscal investment in geological exploration in priority provinces and priority minerals can promote the rational allocation of financial resources for geological exploration.

## References

[1] Ministry of Land and Resources of the People's Republic of China (2000–2010) China Land and Resources Statistical Yearbook. Beijing: Geological Publishing House. pp. 19–226.

[2] Wang W, Wang X, Zhang R (2011) Geological work management system and operation mechanism. Beijing: Geological Publishing House. pp. 6–7.

[3] Ministry of Land and Resources of the People's Republic of China (2010) MLR's proposals on establishing the new mechanism of exploration investment. Available: http://www.mlr.gov.cn/zwgk/zytz/201004/t20100429_717353.htm. Accessed 2013 January 26.

[4] Fang M, Wen ZY (2000) An Analysis of the Geological Prospecting Expense Input and Its Effect. GEOLOGICAL TECHNOECONOMIC NANAGEMENT 1: 26–33.

[5] Wang W, Chen Y, Zhang R (2006) THE APPRAISAL RESEARCH OF THE GEOLOGICAL SURVEY BENEFIT. China Mining Magazine 15: 37–40.

[6] Bing F, Ling-li Z (2011) The research of China mining choice of financing channels. Electronics, Communications and Control (ICECC), 2011 International Conference on IEEE. pp.1138–1141.

[7] Yao HJ, Wang W, Zhang RL (2005) Analysis on Geological Survey Investment and Some Suggestions. Natural Resource Economics of China 9: 29–31, 34.

[8] Liu Y, You X, Gao L, Cheng J (2008) Research on Input-output and Performance Evaluation of Nonprofit Mineral Geological Work. Natural Resource Economics of China 6: 24–28.

[9] Suxun Chenjunnan (2008) Private capital: what impedes its entry into China's minerals industry. Resources Policy 33: 23–28.

[10] Chaissie J, Gugler P (2009) Expansion of Trade and FDI in Asia: Strategic and Policy challenges. New York: Routledge Contemporary Asia Series.pp.101–102.

[11] Zhang X (2011) Probe into Opening Financial Schemes of the Mining Cities Ecological Construction in China. Energy Procedia 13: 1039–1043.

[12] Zhong Z (2002) The Chinese western development initiative: new opportunities for mineral investment. Resources policy 28: 117–131.

[13] Keynes JM (1936) The general theory of employment, interest and money. London: Atlantic Books. pp. 23–29.

[14] Chandra A, Thompson E (2000) Does Public Infrastructure Affect Economic Activity? Evidence from the rural interstate highway system. Regional Science an Urban Economics 30: 457–490.

[15] Arrow KJ, Kurz M (1970) Public investment, the rate of return, and the optimal fiscal policy. Baltimore: Resources for the Future.87 p.

[16] Barro RJ (1990) Government spending in a simple model of endogenous growth. Journal of Political Economy 98: 103–125.

[17] Boarnet MG (1997) Infrastructure Services and the Productivity of Public Capital: the case of Streets and Highway. National Tax Journal 50: 122–134.

[18] Aschauer DA (1989a) Is public Expenditure Productive? Journal of Monetary Economics 23: 177–200.

[19] Tanaka J (2003) Welfare Analysis of a Fiscal Reconstruction Policy in an Overlapping Generations Economy with Public Investment. Journal of Economics 79: 19–39.

[20] Pevcin P (2004) Economic output and Optimal Size of Government. Economic and Business Review 6: 213–227.

[21] Barro RJ (1990) Government spending in a simple model of endogenous growth. Journal of political economy 98: 103–125.

[22] Gunalp B, Guzhan CD (2005) The optimal government size in transition countries. Available: http://ssrn.com/abstract=832605. Accessed 2013 January 26.

[23] Cassou SP, Lansing KJ (1998) Optimal Fiscal Policy, Public Capital, And the productivity slowdown. Journal of economic dynamics and control 22: 911–935.

[24] Karras G (1996) The optimal government size: Further international evidence on the productivity of government services. Economic inquiry 34 2: 193–203.

[25] Karras G (1997) On the optimal government size in Europe: Theory and empirical evidence. Manchester school of Economic & Social Studies 65: 280–294.

[26] Changge YU (2006) An analysis of Economic Effect of Government Public Investment. Journal of Finance and Economics 32: 30–41.

[27] Liu HJ (2009) An Analysis into The Opitmal Scale of Fiscal Income and Expenditute on The Stable Path of China's Economic Growth. Journal of Central University of Finance & Economics 2: 6–10.

[28] Dong XY (2000) Public Investment, Social Services and Productivity of Chinese Household Farms. The Journal of Development Studies 36: 100–122.

[29] Liu Y (2009) Research on appropriate scale estimate of China's government spending and dynamic optimal path. Chinese Journal of Management 6: 1653–1656.

[30] Lin Y (1994) On economic theory of institutional change: induced institutional change and the mandatory regime change. Property rights and institutional change-Proceedings of property rights school and the new system school. Shanghai: Shanghai Joint Publishing.394p.

[31] Zhang J (1996) Optical Public Investment in Education and Endogenous Growth. Scandinavian Journal of Economics 98: 387–404.

[32] Zhong Z, Rao X (2006) Does Optimal size curve of government exist in China? Finance and Trade Research 6: 44–48.

[33] Zhang Z, Hou B, Yao C (2007) Economic growth and the optimal size of government spending - based on the study of national utility function. Statistics and Decision 22: 42–44.

[34] Li X (1996) Financial Reforms and Regional Investment Conflicts in China: A Game-theoretic Analysis. Economics of Planning 29: 117–130.

[35] Wei Y (1996) Fiscal Systems and Uneven Regional Development in China, 1978–1991. GEOFORUM 27: 329–344.

[36] Ma S, Sun C (2005) Study on economic growth and optimal fiscal size. Statistic Research 1: 15–22.

[37] Wang L, Gao W (2011) Nonlinear Effects of Fiscal Policy on Private consumption: Evidence from China. China & World Economy 19: 60–76.

[38] Zhang R, Li Z(2008) Optimal Scale of the Rural Fiscal Expenditure Based on Economic Growth in China. 15th Annual Conference Proceedings of International Conference on IEEE.pp.1197–1202.

[39] Yu Y, Zhang L, Li F, Zheng X (2011) On the determinants of public infrastructure spending in Chinese cities: A spatial econometric perspective. The Social Science Journal 48: 458–467.

[40] Allen F, Qian J, Zhang C, Zhao M (2012) China's Financial System: Opportunities and Challenges. No. w17828. National Bureau of Economic Research.pp.3–10.

[41] Huang H (2011) Decentralization Orientation and Principles of Perfecting Fiscal System below Province Level in China. Journal of Guangxi University of Finance and Economics 1: 12.

[42] Li XU, Chun WAN (2010) Path Dependence and Institutional Innovation in the Evolution of China's Fiscal System. Technoeconomics & Management Research 4: 27.

[43] Guo Q, Jia J (2010) Fiscal Decentralization, Government Structure and Local Government's Expenditure Size. Economic Research Journal 11: 8.

[44] Guo Q (2012) The Incentive Effects of the Institutional Arrangements of China's Fiscal System and Their Influence on Finance and Economics. Social Sciences in China 33: 104–107.

[45] Wong C, Bird R (2008) China's fiscal system: a work in progress. China's Great Economic Transformation. pp. 429–466.

[46] Zhang X (2006) Fiscal decentralization and political centralization in China: Implications for growth and inequality. Journal of Comparative Economics 34: 713–726.

[47] Zhan JV (2009) Decentralizing China: analysis of central strategies in China's fiscal reforms. Journal of Contemporary China 18: 445–462.

[48] Besley T, Coate S (2003) Centralized versus decentralized provision of local public goods: a political economy approach. JOURNAL OF PUBLIC ECONOMICS 87: 2611–2637.

[49] Jan H, Evzen K (2011) Public Investment and Fiscal Performance in the New EU Member States. FISCAL STUDIES 32: 43–71.

[50] Wen J (2009) Report on the Work of the Government. delivered at the second session of the Eleventh National People's Congress. pp. 24–25.

[51] Remmer KL, Wibbels E (2000) The subnational politics of economic adjustment - Provincial politics and fiscal performance in Argentina. COMPARATIVE POLITICAL STUDIES 33: 419–451.

[52] Anderson GJ (1981) A new approach to the empirical investigation of investment expenditures. The Economic Journal 91: 88–103.

[53] Bean CR (1981) An econometric model of manufacturing investment in the UK. The Economic Journal 91: 106–121.

[54] Bügel MS, Buunk AP, Verhoef PC (2010) A comparison of customer commitment in five sectors using the psychological investment model. Journal of Relationship Marketing 9: 2–29.

[55] Collins LR (2011) College females' decisions to stay or leave an abusive relationship: a test of the investment model. Diss. University of North Carolina at Greensboro. pp. 4–9.

[56] Derlega VJ, Winstead BA, Pearson MR, Janda LJ, Lewis RJ, et al (2011) Unwanted Pursuit in Same-Sex Relationships: Effects of Attachment Styles, Investment Model Variables, and Sexual Minority Stressors. Partner Abuse 2: 300–322.

[57] Fischer S, Frenkel JA (2012) Investment, the two-sector model and trade in debt and capital goods. Journal of International Economics 2: 211–233.

[58] Hines AG, Catephores G (1970) Investment in UK manufacturing industry, 1956–67. The econometric study of the United Kingdom. London: Macmillan and Co., Ltd. pp. 202–224.

[59] Linna L, Yalin L, Ge J (2012) Economic impact measurement and evaluation of China's investment in geological exploration: The empirical analysis based on the data from 1999 to 2009. Resources Policy 37: 375–384.

[60] Chinese National People's Congress (2011) People's Republic of China's implementation of the national budget in 2010 and national budget in 2011 (Draft). Available: http://www.npc.gov.cn/wxzl/gongbao/2011-08/16/content_1665647.htm. Accessed 2013 January 26.

