# Correction: Scale Determinants of Fiscal Investment in Geological Exploration: Evidence from China

**DOI:** 10.1371/annotation/036be06c-69f9-4b4d-a895-d16c76611330

**Published:** 2014-01-02

**Authors:** Linna Lu, Yalin Lei

The affiliation of the second author is incorrect. Yalin Lei is not affiliated with institution number 2, but rather with institution number 1, School of Humanities and Economic Management, China University of Geosciences, Beijing, China. 

